# Supplementary material for: Posterior Reversible Encephalopathy Syndrome in Children with Malignancies or After Hematopoietic Cell Transplantation: A Polish Nationwide Study
Source: Cancers (Basel). 2025 Nov 26;17(23):3789. doi: 10.3390/cancers17233789 (PMC12691463; doi:10.3390/cancers17233789)
Supplement: Supplementary file 1 [file cancers-17-03789-s001.zip › cancers-3998111-supplementary.pdf]

cancers

MDPI

Supplementary Materials: Posterior Reversible Encephalopathy Syndrome in Children with Malignancies or After Hematopoietic Cell Transplantation: A Polish Nationwide Study

Tomasz Brzeski, Wanda Badowska, Katarzyna Mycko, Patrycja Tyszka, Martyna Korzeniewicz, Julia Kolodrubiec, Wojciech Mlynarski, Karolina Gawle-Krawczyk, Katarzyna Koch, Pawel Laguna, Aleksandra Kiermasz, Agnieszka Mizia-Malarz, Marta Malczewska, Katarzyna Drabko, Anna Malecka, Ninela Irga-Jaworska, Patrycja Marciniak-Stepak, Katarzyna Derwich, Jacek Wachowiak, Magdalena Bartnik, Tomasz Ociepa, Tomasz Urasinski, Malgorzata Sawicka-Zukowska, Maryna Krawczuk-Rybak, Grzegorz Waliszczak, Walentyna Balwierz, Szymon Skoczen, Tomasz Jarmolinski, Krzysztof Kalwak, Iwona Ruranska, Tomasz Szczepanski, Wioletta Bal, Radosław Chaber, Magdalena Tarasinska, Bozenna Dembowska-Baginska, Agnieszka Chodala-Grzywacz, Grazyna Karolczyk, Sonia Pajak, Monika Richert-Przygonska, Krzysztof Czyzewski and Jan Styczynski

Table S1. Characteristics of study group vs. control group with p adj.

Parameter	Study group (PRES)	Control group (non-PRES)	p	p adj
Number of patients (%)	120 (100.0)	318 (100.0)	-	-
Female [n (%)]	44 (36.7)	137 (43.1)	0.268	0.421
Male [n (%)]	76 (63.3)	181 (56.9)	0.268	0.421
Week of gestation 37-42* [n (%)]	65 (90.3)	186 (89.9)	> 0.999	> 0.999
Apgar < 8 pts.* [n (%)]	1 (1.4)	4 (2.0)	> 0.999	> 0.999
Epilepsy before malignancy [n (%)]	6 (5.0)	3 (0.9)	0.015	0.045
Hypertension before malignancy [n (%)]	5 (4.2)	3 (0.9)	0.039	0.081
Hypertension in the family [n (%)]	5 (4.2)	10 (3.1)	0.567	0.744
Developmental delay [n (%)]	6 (5.0)	18 (5.7)	0.972	> 0.999
Age at diagnosis, years, [M ± SD]	7.99 ± 3.77	6.89 ± 4.54	0.019	0.049
Treatment for relapse	17 (14.2)	24 (7.5)	0.053	0.099
Age at relapse, years, [M ± SD]	10.11 ± 4.82	10.56 ± 4.55	0.808	0.943
HCT [n (%)]	10 (8.3)	36 (11.3)	0.462	0.647
Age at HCT, years, [M ± SD]	12.58 ± 4.68	10.66 ± 4.56	0.281	0.421
Disease			0.057	0.099
Acute lymphoblastic leukemia [n (%)]	92 (76.7)	273 (85.8)		
Acute myeloid leukemia	4 (3.3)	9 (2.8)		
Other malignancies [n (%)]	24 (20.0)	36 (11.3)		
CNS involvement [n (%)]	23 (19.2)	53 (16.7)	0.635	0.784
Hypertension during treatment [n (%)]	89 (74.2)	45 (14.2)	< 0.001	< 0.001
ICU admission [n (%)]	60 (50.0)	94 (29.6)	< 0.001	< 0.001
Seizures during treatment [n (%)]	96 (80.0)	21 (6.6)	< 0.001	< 0.001
Hypertension during follow-up	27 (22.5%)	45 (14.2)	0.035	0.081
Epilepsy during follow-up	25 (20.8%)	0**	<0.001	< 0.001
Remission at the end of follow-up [n (%)]	92 (76.7)	298 (93.7)	< 0.001	< 0.001
Death [n (%)]	25 (20.8)	21 (6.6)	< 0.001	< 0.001

* The percentage is given in relation to all patients with information on the week of gestation (study group: n = 72, control group: n = 207) and Apgar score (study group: n = 71, control group: n = 204). **Data available for 189 patients from the control group. p adj – outcomes after the correction for multiple comparisons, made with Benjamini-Hochberg method.

 https://doi.org/10.3390/cancers17233789

Table S2. Types of disease in particular laboratory findings during PRES prodromal period – study group.

Parameter	ALL	NHL	Others
Elevated CRP (n=62)	49	7	6
Electrolyte disturbances (n=90)	69	13	8
Hypertransaminasemia (n=52)	37	6	9
